# Acute rectal calcinosis following parathyroidectomy in a patient with primary hyperparathyroidism and preoperative hypercalcaemic crisis

**DOI:** 10.1530/EDM-25-0100

**Published:** 2025-12-09

**Authors:** Helēna Apele, Dace Seisuma

**Affiliations:** ^1^ Rīgas Stradiņš University, Riga, Latvia; ^2^ Pauls Stradiņš Clinical University Hospital, Riga, Latvia

**Keywords:** primary hyperparathyroidism, rectal calcinosis, parathyroidectomy, hypercalcaemic crisis, pituitary

## Abstract

**Summary:**

This case highlights a potentially underrecognised complication – acute rectal calcinosis with ulceration in the context of parathyroidectomy due to primary hyperparathyroidism (PHPT), which is a common endocrine disorder characterised by excessive secretion of parathyroid hormone (PTH), most often due to a solitary adenoma of the parathyroid gland. While severe hypercalcaemia is a known complication, its association with visceral metastatic calcifications, particularly in the rectum, is extremely rare and has not previously been reported. We describe a case of a 73-year-old female who presented with progressive symptoms of hypercalcaemia, including significant weight loss, gastrointestinal involvement, and lethargy. After repeated emergency department attendances, a hypercalcaemic crisis was confirmed. Despite initial rehydration and supportive therapy, her condition deteriorated, requiring hemofiltration. Further evaluation confirmed PHPT, revealing markedly elevated intact parathormone (iPTH) and a 5 cm parathyroid adenoma, which was removed surgically. The day following parathyroidectomy, the patient developed acute rectal bleeding with a significant haemoglobin drop. Computed tomography imaging showed rectal wall thickening and hyperdensity, while colonoscopy revealed deep rectal ulcerations with visible calcifications and inflammation. Conservative treatment, including blood component transfusions, electrolyte management, and local haemostasis, led to gradual clinical improvement.

**Learning points:**

- Severe hypercalcaemia can lead to visceral calcifications, ischaemic changes, and gastrointestinal complications, even if not initially evident.
- In patients with primary hyperparathyroidism, acute rectal bleeding following parathyroidectomy may indicate a rare cause such as rectal calcinosis and should be considered in the differential diagnosis.
- Calcinosis should be particularly suspected in cases with a severe clinical picture of hyperparathyroidism-related hypercalcaemia (e.g. severe weight loss, lack of calcium correction after standard therapy).
- Early diagnosis and conservative management can result in favourable outcomes without the need for invasive treatment.

## Background

This case presents a poorly documented complication of primary hyperparathyroidism (PHPT), manifesting as acute rectal calcinosis with ulceration, most likely triggered by hypercalcaemia and the rapid mobilisation of minerals post-parathyroidectomy. According to current knowledge, rectal calcinosis might develop in a small subgroup of patients with primary hyperparathyroidism, and additional risk factors might be involved (e.g. age, previously diagnosed or undiagnosed digestive disorders, or exposure to certain medication). Such a condition should be included in the differential diagnosis of rectal bleeding in patients with PHPT, particularly following surgery.

In addition, conservative management is important for addressing calcified lesions, as demonstrated by the fact that surgical intervention was unnecessary, and the calcifications resolved spontaneously over time.

## Case presentation

A 73-year-old female patient has a medical history of grade 2 primary arterial hypertension, which was treated with the angiotensin-converting enzyme inhibitor (perindopril) and the calcium channel blocker (amlodipine). The patient’s family history revealed no conditions directly related to hyperparathyroidism.

At her first presentation to the general practitioner, the patient reported symptoms of excessive thirst, diminished appetite, and gradual unintentional weight loss of 20 kg in 8 months, as well as normochromic normocytic anaemia, which was not further investigated. A few months later, her condition progressively worsened, with the development of fatigue, extreme frailty, hypochromic microcytic anaemia, generalised weakness, confusion, constipation, nausea, vomiting, abdominal pain, dehydration, and acute kidney injury.

On the emergency department visit, laboratory results revealed a hypercalcaemic crisis with calcium levels 5.2 mmol/L (reference range (RR): 2.2–2.6 mmol/L). She was admitted to the intensive care unit and managed with rehydration and supportive therapy. The same day, the patient was transferred to the endocrinology department, where a diagnosis of PHPT was made with an intact parathormone (iPTH) level of 1675 pg/mL (RR: 15–65 pg/mL).

Hyperparathyroidism of renal origin was considered unlikely despite mildly impaired renal function (creatinine 122 μmol/L, RR: 49–90; eGFR 40 mL/min) with urea at the upper normal limit (7.8 mmol/L, RR: 2.5–7.8 mmol/L), as the patient had no history of chronic kidney disease and serum phosphate was within the normal range (0.98 mmol/L, RR: 0.78–1.65), further favouring a primary aetiology of hyperparathyroidism. The diagnosis was confirmed following contrast-enhanced neck ultrasound, which revealed a 5 cm hypoechoic, irregular, hypovascularised tumour in the right upper parathyroid gland.

As calcium levels remained critically high despite calcium-lowering therapy (rehydration and loop diuretics), and given the patient’s severe clinical condition and the fact that denosumab and bisphosphonates were not administered based on the treating physician’s clinical judgement (as will be addressed in the Discussion section), haemofiltration was performed. Given the severity of the case, 8 grams of chief cell adenoma was surgically removed. Postoperatively, the patient suddenly developed rectal bleeding due to calcinosis.

During hospitalisation, the following conditions were identified: osteopenia (lumbar spine T-scores: −1.8 and −2.1 SD; femoral: −2.1 SD), nephrolithiasis, a multinodular euthyroid goitre, a right adrenal incidentaloma, and a right ovarian cyst.

## Investigation

**Figure 1:**
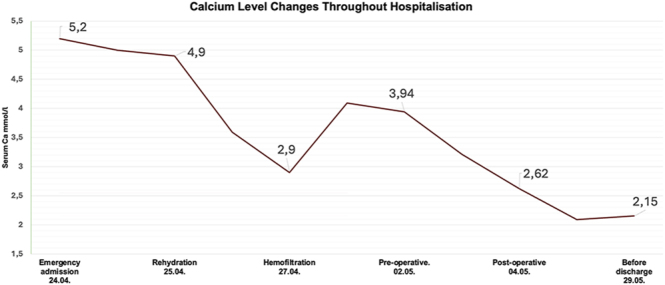
Serum calcium level changes throughout hospitalisation, from emergency admission to discharge.

Postoperative bleeding led to a severe haemoglobin drop from 119 to 63 g/L (RR: 120–160 g/L for females). The patients’ inflammatory markers were also elevated. Despite haemofiltration resulting in a notable decrease in calcium levels, during hospitalisation it remained high, and only after the selective parathyroidectomy and further substitution therapy did calcium normalise (Fig. 1). The same was true for iPTH, which normalised to 34 pg/mL.

**Figure 2:**
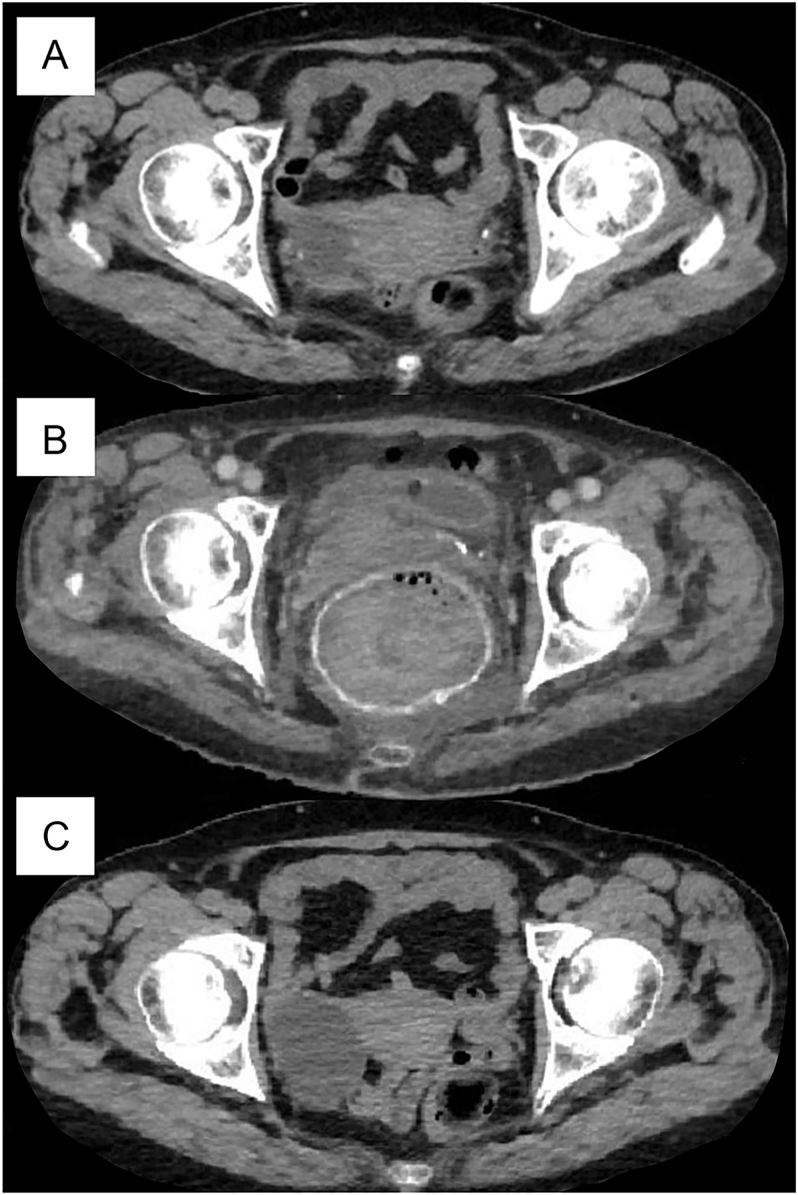
Abdominal CT imaging: almost 3 weeks before the surgery (A), the next day after parathyroidectomy (B), and 2 weeks after parathyroidectomy (C).

An urgent computed tomography (CT) scan with intravenous contrast revealed marked rectal dilation (8 cm diameter) with hyperdense walls (230 Hounsfield units) that was filled with content (Fig. 2). CT of the brain and chest was unremarkable for calcifications. Interestingly, an abdominal CT scan performed nearly 3 weeks before hospitalisation – unrelated to rectal symptoms – showed no pathological rectal changes, suggesting the rapid onset of calcinosis (Fig. 2). Moreover, follow-up imaging demonstrated surprisingly fast resolution of the rectal calcinosis within just a few weeks (Fig. 2), indicating a fast resorption process.

**Figure 3:**
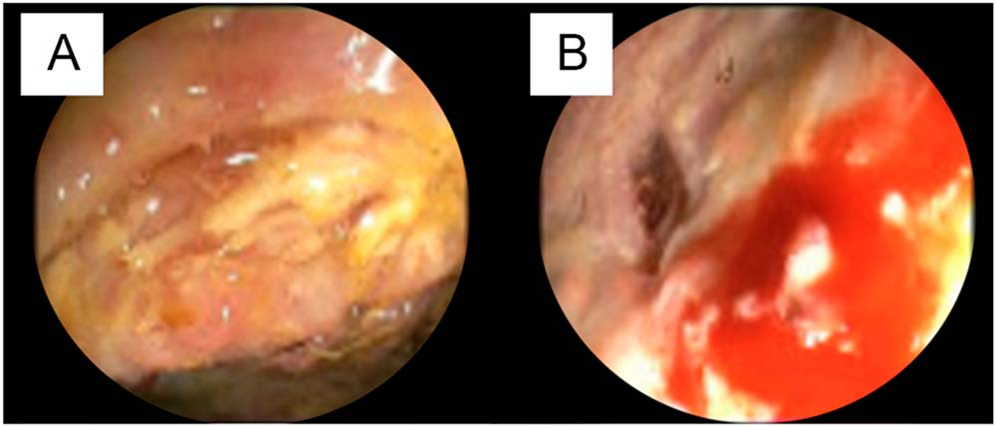
Colonoscopy images showing pale, firm calcified deposits at the ulcer base (A) and irregular, extensive ulceration with epithelial detachment and focal bleeding (B).

Multiple colonoscopies confirmed an irregular, extensive ulceration with epithelial detachment. The ulcer bases contained a pale, firm substrate suggestive of calcifications. Focal oozing of blood was observed (Fig. 3).

A few days later, histopathological evaluation of rectal biopsy (from ulcer margins) showed a chronically active ulcer composed of fibrin, debris, calcium deposits, neutrophilic leukocytes, and focal mucosal necrosis. No evidence of malignancy was found in the examined tissue.

## Treatment

Parathyroidectomy was performed on the fifth day of hospitalisation, followed by supplementation with calcium (calcium carbonate 1,000 mg three times daily) and active vitamin D (calcitriol initially 1 μg, subsequently titrated to 0.25 μg twice daily).

Before the surgery, the patient received medical therapy, including oral cinacalcet, oral potassium chloride, intravenous dexamethasone, isotonic fluids (0.9% sodium chloride, Ringer’s solution), and loop diuretics (intravenous furosemide, oral torasemide).

Upon onset of rectal bleeding, conservative management was initiated, which included red blood cell and fresh frozen plasma transfusions, rectal haemostatic treatment (intravenous ethamsylate, argon plasma coagulation), continued intravenous fluids, and loop diuretics.

## Outcome and follow-up

**Figure 4:**
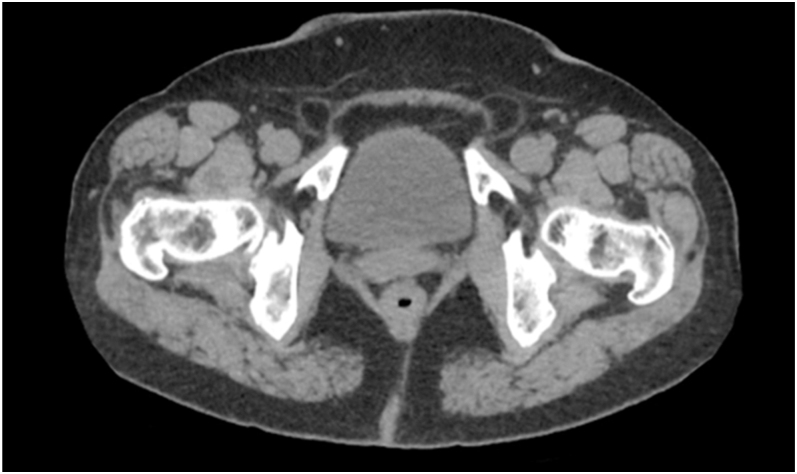
Abdominal CT almost 2 years after parathyroidectomy, without intestinal abnormalities.

During the first 2 weeks following the initial bleeding episode, the patient experienced persistent mild rectal bleeding, which gradually resolved over time. Serial imaging showed gradual resolution of rectal wall changes after conservative treatment. With ongoing calcium and vitamin D supplementation, serum calcium and iPTH normalised postoperatively and remained stable, with improved renal function (serum creatinine decreased to 67 μmol/L, corresponding to an estimated GFR of approximately 74 mL/min/1.73 m^2^). Haemoglobin increased to 92 g/L and inflammatory markers decreased. The patient recovered without the need for surgical intervention and was discharged in stable condition. Follow-up after 2 years showed no evidence of recurrence (Fig. 4).

## Discussion

Primary hyperparathyroidism (PHPT) is a relatively well-recognised endocrine disorder among clinicians, particularly due to its characteristic laboratory findings such as hypercalcaemia and elevated parathyroid hormone (PTH) levels (1). However, despite its familiarity, certain aspects of PHPT remain insufficiently explored, especially those concerning the acute metabolic consequences following parathyroidectomy. In this case, our patient, who had experienced long-standing, nonspecific symptoms, developed a rare and rapidly progressing complication – rectal bleeding associated with calcinosis – shortly after the surgery. Notably, prior imaging did not reveal any signs of intestinal pathology, which raises questions about the underlying mechanisms that could have led to such abrupt calcium deposition in the rectal tissue.

A rational hypothesis for rectal calcinosis following a hypercalcaemic crisis and parathyroidectomy involves a sudden disturbance in calcium-phosphate homoeostasis, triggering ectopic calcification processes. According to recent studies on mechanisms of action, soft tissue calcification is not merely passive but a highly regulated phenomenon involving apoptotic cell release of matrix vesicles and transdifferentiation of resident cells into osteoblast-like phenotypes, particularly in tissues affected by microdamage, chronic low-grade inflammation, or reduced perfusion (2). In our case, even without evidence of atherosclerosis, previous hypercalcaemia may have led to microvascular impairment and ischaemia in the rectal tissue, thereby creating a vulnerable environment conducive to dystrophic calcification (2). In addition, episodes of constipation could have further exacerbated local tissue stress and hypoxia, fostering conditions favourable for mineral deposition. While the exact pathogenesis of rectal calcinosis remains unclear due to limited clinical data, this case underscores the need to investigate the role of calcium-phosphate imbalance, local tissue damage, and inflammation in soft tissue mineralisation after parathyroidectomy.

Soft tissue calcium salt deposition beyond vascular and dermal compartments is well‐documented in metabolic and hereditary syndromes (3), yet gastrointestinal involvement, especially post-parathyroidectomy, is exceedingly rare. Literature on postoperative ectopic calcification in this setting remains scarce, and its pathophysiology is not well understood. Depending on tumour location, outcomes can range from mild functional impairment to life-threatening haemorrhage or obstruction (3). This clinical rarity highlights the need for targeted research to identify risk factors and guide perioperative calcium-phosphate management, including potential therapies to modulate mineral metabolism.

In our case, bisphosphonates were withheld preoperatively due to concern for hungry bone syndrome (HBS), marked postoperative hypocalcaemia from rapid skeletal remineralisation (4). The patient had known HBS risk factors: older age, large adenoma, and markedly elevated preoperative calcium and PTH levels (4). Coexisting acute kidney injury also raised concern for bisphosphonate-induced nephrotoxicity, as these agents are renally excreted (5). However, retrospective studies suggest bisphosphonates may actually lower HBS risk by tempering abrupt bone turnover after surgery (4). Furthermore, the patient’s osteopenia requires attention; without appropriate therapy (e.g. bisphosphonates or denosumab), the risk of fracture may increase (6). Thus, cautious preoperative bisphosphonate use, alongside calcium and vitamin D support, might have been both safe and beneficial.

Timely recognition and initiation of conservative management are crucial to prevent serious outcomes, such as severe tissue damage or the need for invasive procedures. Although PHPT is common, its complications can be unexpectedly complex. This case emphasises the importance of accurate diagnosis and vigilance for rare postoperative complications, even after successful treatment of the underlying disorder.

## Declaration of interest

The authors decalre that there is no conflict of interest that could be perceived as prejudicing the impartiality of the research reported.

## Funding

This research did not receive any specific grant from any funding agency in the public, commercial, or not-for-profit sector.

## Patient consent

Written informed consent for publication of clinical details and clinical images was obtained from the patient.

## Author contribution statement

HA was responsible for the collection of clinical data, literature review, and writing of the manuscript. DS supervised the case, verified the clinical content, and provided critical revisions to the final version of the manuscript. DS is the treating physician of the patient and gave permission to report this case.

## Patient’s perspective

The patient described that she now feels well. She reported being able to lead an active daily life, although she continues to experience fatigue and has cardiovascular comorbidities, including arrhythmia, episodes of tachycardia, and fluctuating blood pressure. Calcium supplementation has been discontinued, and her laboratory results have remained within normal limits. She also noted that she has regained the weight that was previously lost. The patient expressed that, before receiving appropriate treatment, her complaints persisted for a long time, and no one was able to provide effective help, which created a significant emotional burden. She specifically emphasised that her condition began to improve only after DS took over her care. Follow-up has shown no signs of recurrence of PHPT and rectal calcinosis.

## References

[1] Pokhrel B & Levine SN. Primary Hyperparathyroidism: PubMed; StatPearls Publishing, 2024. (https://www.ncbi.nlm.nih.gov/books/NBK441895/) 28722924

[2] Proudfoot D. Calcium signaling and tissue calcification. Cold Spring Harbor Perspect Biol 2019 11 a035303. (10.1101/cshperspect.a035303) PMC6771368 31138543

[3] Snijders BMG, Peters MJL & Koek HL. Ectopic calcification: what do we know and what is the way forward? J Clin Med 2023 12 3687. (10.3390/jcm12113687) 37297880 PMC10253322

[4] Cartwright C & Anastasopoulou C. Hungry Bone Syndrome. StatPearls Publishing, 2022. (https://www.ncbi.nlm.nih.gov/books/NBK549880/) 31751070

[5] Torregrosa J & Ramos A. Uso de bifosfonatos en la enfermedad renal crónica. Madrid Nefrologia 2010 30 288–296. (10.3265/Nefrologia.pre2010.Mar.10320) 20514097

[6] Bandeira F, de Moura Nóbrega J, de Oliveira LB, et al. Medical management of primary hyperparathyroidism. Arch Endocrinol Metab 2022 66 689–693. (10.20945/2359-3997000000558) 36382758 PMC10118813

